# The Derbyshire Royal Infirmary

**Published:** 1895-03-09

**Authors:** 


### HOSPITAL ADMINISTRATION.

### THE DERBYSHIRE ROYAL INFIRMARY.

The Position of the New Members of the Honorary Medical Staff.

By E. Collier Green, L.R.C.P. Lond., M.R.C.S., Honorary Secretary to the Honorary Medical Staff.

I do not think that your correspondent, from whose letter you quote on February 16th, has given you a fair account of the action of the governors of the Derbyshire Royal Infirmary *re* the new appointments on the honorary medical staff.

Briefly stated, the facts are as follows:—

Under the new rules, it was at first proposed to appoint one assistant physician and one assistant surgeon, in addition to the present staff of three physicians, three surgeons, and one ophthalmic surgeon. As, however, it was found that some governors objected to the word "assistant," the committee reconsidered the subject, and agreed to withdraw the word "assistant," but left the duties of the new officers as at first proposed. In other words, it was agreed that the two new members of the staff should at once be given the full title of physician and surgeon, but that they should perform the duties usually performed by assistant physicians and surgeons, until such time as there should be vacancies on the full staff. This proposal was brought before a meeting of the governors. One governor proposed as an amendment that the two new members of the staff should share the beds equally with the other members of the staff; this found no seconder, and the original proposition was carried unanimously.

To say that the new officers are "underlings of the present staff" is a statement which is not borne out by fact. It is certainly proposed that they shall have charge of beds when other members of the staff are away, but this very usual arrangement is generally considered rather as a privilege than as a hardship.

Does it add to the efficiency of a hospital to divide the beds between too many officers? At present the number of beds allotted to each of the staff is much below the average in provincial hospitals, and if the suggested proposal were adopted, it would reduce the number of beds per officer below that of any important hospital in the country.

The present members of the staff have worked hard, and have spared neither time nor trouble to render the hospital as perfect as possible, both as regards the new building and the medical and surgical work done; surely it would be scarcely fair for the governors to now take away a fourth of their beds from them.

From the enclosed list of provincial hospitals, you will see that it is not unusual to have one or more assistant physicians and surgeons attached to the staff, and that in no case, except in one hospital of 210 beds, are there four full physicians and four full surgeons.

I send you our reports for the last three years, and if you will turn to the tables of the number of patients, the diseases of in-patients and of the operations performed, I do not think that you will share the fear of your correspondent as to "the reduction of this fine institution to the level of a workhouse infirmary," or "a mere resting-place for chronic cases."

You very properly emphasise the importance of the out-patient department, by attachment to which no physician or surgeon need think himself degraded. Only a moiety of this work will devolve on the new officers, the remainder being still undertaken by the present members of the staff.

The reasons which led "the only F.R.C.S. in the town" to decline] the office (or rather to decline to compete for the office) are best known to himself, but it is scarcely possible to believe that any surgeon can seriously maintain that the office, as proposed, is anything but a strictly honourable and professional one, and one in which he could not do much good, and render himself still more skilful in his art. When the time should come for him to share the beds, he would be glad that they were divided between three instead of between four surgeons.

I may say that it is by no means true that the better class of the inhabitants always go outside the town when they want a consultant; the infirmary staff get a very fair amount of consultation work.

⁂ We would suggest that the new members of the honorary medical staff should have the titles of "physician in charge of out-patients" and of "surgeon in charge of out-patients" respectively. This would get over the difficulty, and be in accordance with precedent.—Ed. *T. H.*

